# Supplementary figures and images for: Comparing T cell receptor repertoires using optimal transport
Source: PLoS Comput Biol. 2022 Dec 7;18(12):e1010681. doi: 10.1371/journal.pcbi.1010681 (PMC9728925; doi:10.1371/journal.pcbi.1010681)

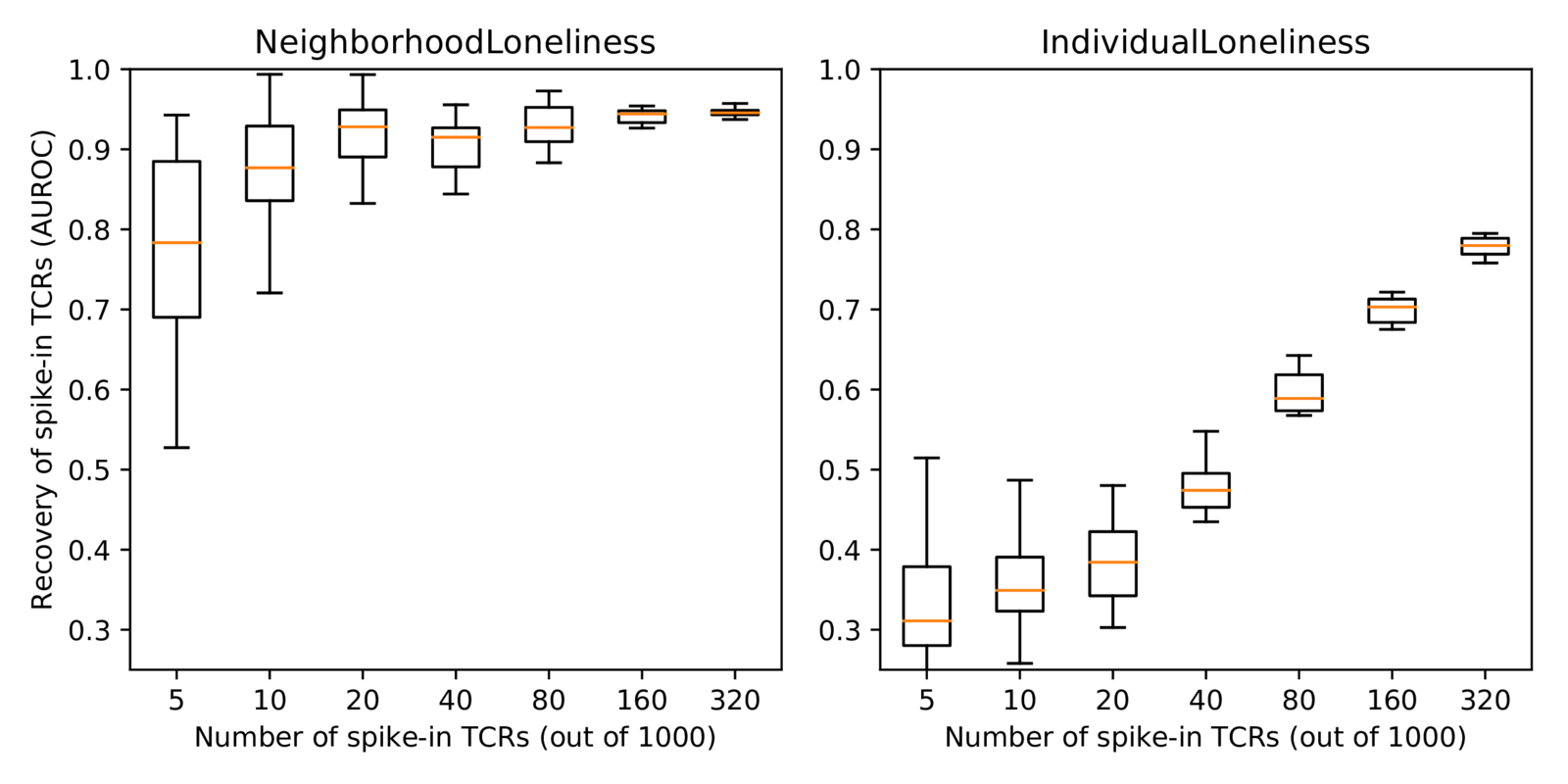

Supplement: S1 Fig — Each bar summarizes the Area Under the Receiver Operating Characteristic curve (AUROC) values for 10 replicate experiments in which a varying number (x-axis) of TCRs sharing a single epitope specificity were spiked into one of two repertoires randomly sampled from a large population of naive CD8+ T cells. The AUROC measures the ability of the corresponding measure (neighborhood loneliness on the left or individual loneliness on the right) to differentiate the spiked-in TCRs from the naive CD8+ TCRs when comparing the two repertoires. (TIFF) [file pcbi.1010681.s001.tiff]

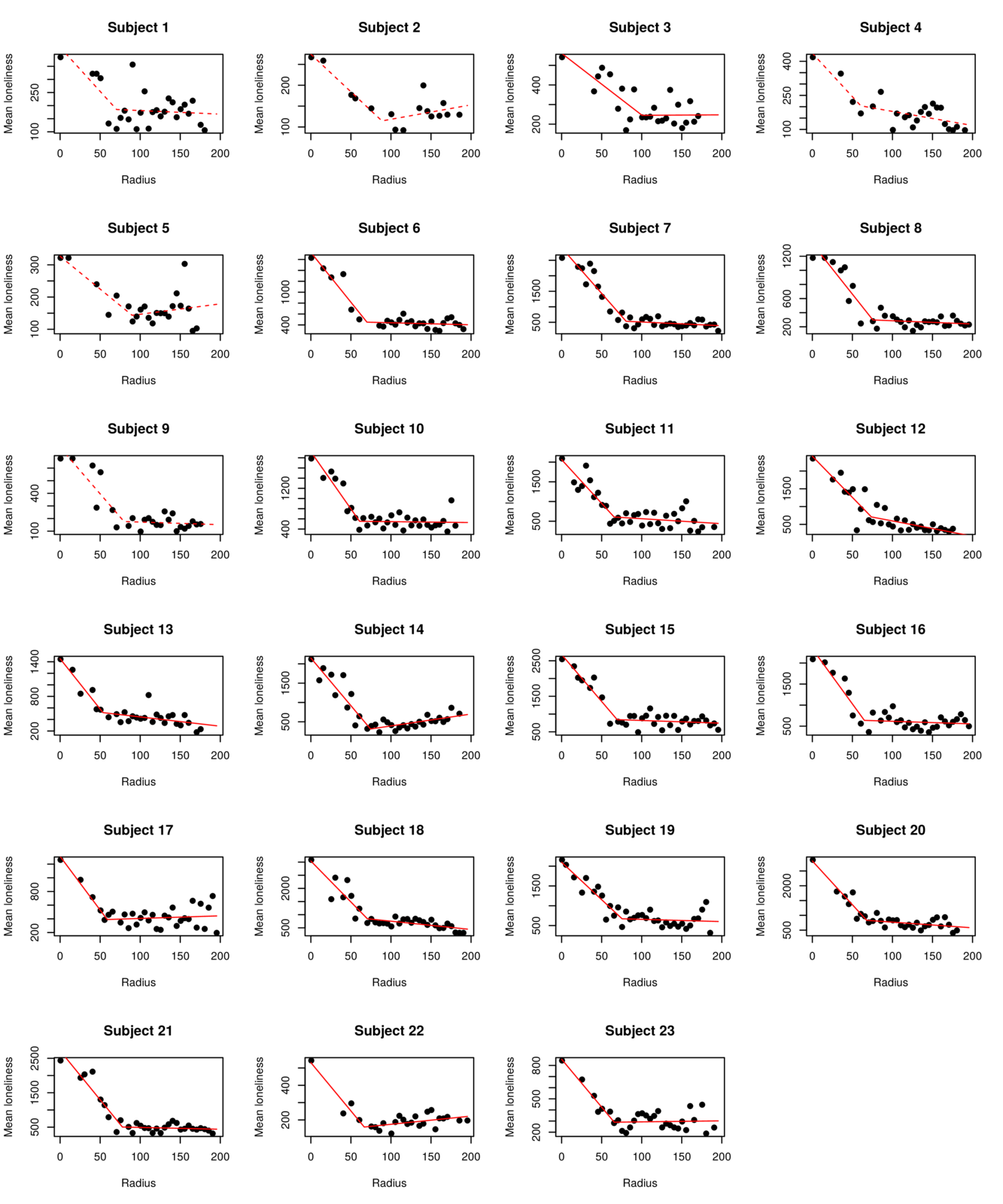

Supplement: S2 Fig — This is a visual check of the assumptions of the segmented regression specified by Eq (14), showing that the assumptions hold in the relationship of mean annulus loneliness versus TCRdist from the centroid, and that this relationship has identifiable breakpoints for a typical repertoire. We perform Algorithm 1 on each full repertoire, which yields the “loneliest” cluster of each repertoire. We see that we are able to successfully estimate a breakpoint rbreakpoint for each subject, with rbreakpoint ∈ (50, 100) for almost all subjects. When the repertoire contains fewer than 200 TCRs, the relationships can weaken (e.g. Subject 1), though Algorithm 1 still provides sensible regression estimates. When the repertoire contains at least 200 TCRs, we see consistent elbow behavior and convincing breakpoint estimates. Furthermore, violations of the least squares assumptions do not appear to be a concern. (TIFF) [file pcbi.1010681.s002.tiff]

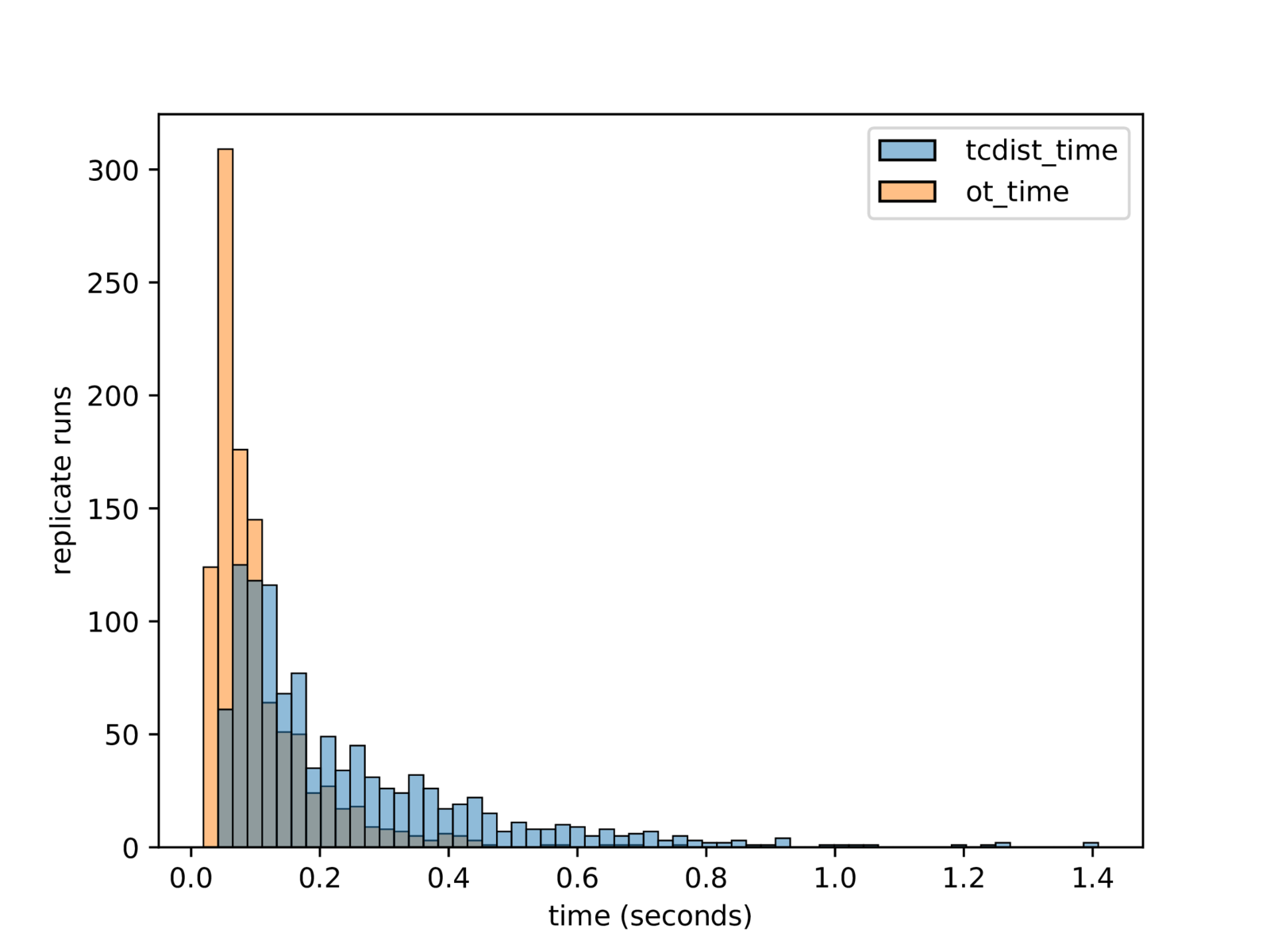

Supplement: S3 Fig — (TIFF) [file pcbi.1010681.s003.tiff]
