# Supplementary material for: Comparing T cell receptor repertoires using optimal transport
Source: PLoS Comput Biol. 2022 Dec 7;18(12):e1010681. doi: 10.1371/journal.pcbi.1010681 (PMC9728925; doi:10.1371/journal.pcbi.1010681)
Supplement: Attachment — Submitted filename: responses.pdf [file pcbi.1010681.s004.pdf]

Responses to reviews for "Comparing T cell receptor repertoires using optimal transport"

Olson et al.

NOTE: Reviewer comments are shown in *blue italic*. Author responses are shown in black. New manuscript text is shown in *orange italic*.

Editor

Thank you very much for submitting your manuscript "Comparing T cell receptor repertoires using optimal transport" for consideration at PLOS Computational Biology. As with all papers reviewed by the journal, your manuscript was reviewed by members of the editorial board and by several independent reviewers. In light of the reviews (below this email), we would like to invite the resubmission of a significantly-revised version that takes into account the reviewers' comments. Your revised manuscript will be sent to the reviewers for further evaluation. They raised several substantial concerns; please address these as carefully as possible, and note that we cannot guarantee acceptance of the revised version. When you are ready to resubmit, please upload the following:

1. A letter containing a detailed list of your responses to the review comments and a description of the changes you have made in the manuscript. Please note while forming your response, if your article is accepted, you may have the opportunity to make the peer review history publicly available. The record will include editor decision letters (with reviews) and your responses to reviewer comments. If eligible, we will contact you to opt in or out.

2. Two versions of the revised manuscript: one with either highlights or tracked changes denoting where the text has been changed; the other a clean version (uploaded as the manuscript file). Important additional instructions are given below your reviewer comments.

Please prepare and submit your revised manuscript within 60 days. If you anticipate any delay, please let us know the expected resubmission date by replying to this email. Please note that revised manuscripts received after the 60-day due date may require evaluation and peer review similar to newly submitted manuscripts.

Thank you again for your submission. We hope that our editorial process has been constructive so far, and we welcome your feedback at any time. Please don't hesitate to contact us if you have any questions or comments.

Thank you very much for your thorough assessment of our manuscript. We have addressed the reviews by

adding in several new analyses as well as many manuscript edits.

We apologize to the reviewers for the long delay in revising our manuscript. The first author has moved onto a position in industry, and has not been able to lead the revision process.

Reviewer 1

Summary: Olson and colleagues present a method for comparing TCR repertoires and detect significant differences between them. Comparison of two repertoires is formulated in terms of a classical computer science problem (discrete optimal transport) with a TCR-specific metric (TCRdist). The authors provide a concise formal definition of this problem and elegantly adapt it to TCR repertoires. They also describe a statistical procedure for testing if the calculated repertoire difference is significant and show how to identify TCRs and motifs that are responsible for the difference. The authors validate this statistical procedure with experimental data from biological replicates and apply their method to longitudinal data from individuals vaccinated against yellow fever to identify post-vaccination shifts in the TCR repertoires. However, the authors did not test their method on ground truth data (simulated) rendering an evaluation of the method difficult. More generally, the figures and test are nearly impossible to understand, Therefore, the real-life application of the method is unclear. The major and minor issues related to paper are discussed below:

Major issues

Biological usefulness: the most biological claim is that "the framework can successfully extract biologically meaningful regions between distinct TCR populations" but the meaning of these regions is unclear.

The reviewer is correct that we used the word "region" loosely when referring to TCR repertoires. We have removed all instances of this term, unless specifically referring to the cartoon in Figure 1 which depicts an abstract "TCR space."

TCRdist is known in the community for high running time. In the current manuscript, the authors avoided running time problems by analyzing relatively small samples: "Each repertoire is filtered to the 1,000 most abundant clones". The robustness of the method to such downsampling needs to be shown, and running time statistics for the computations in the manuscript need to be provided. Specifically, it would be great to see simulations to understand the sensitivity of the method.

We have included a comparison (Fig. S3) of the runtime required for the optimal transport calculation compared to the calculation of TCRdist, which shows that in practice runtime is dominated by the TCRdist

calculation, even with a faster C++ implementation than the original.

Regarding sensitivity, thank you for the suggestion. We have included a simulation study in which varying numbers of "spike-in" epitope-specific TCRs are added to one of two random samplings from a diverse CD8 repertoire and our ability to discriminate these spiked-in TCRs is examined. This can be found as "Simulation-based benchmarking" as the last section before the Discussion.

AIRR analyses often tend to be sensitive to data preprocessing (clonotype computation etc.). Does the described method require for both repertoires under comparison to be preprocessed in an identical way? Will the comparison conclusion hold if both datasets are first processed identically in one way, and then identically another way (e.g. with different preprocessing tool parameters). Generally, robustness of the described method to preprocessing differences needs to be shown or at least explained.

By virtue of being based on amino acid differences, we believe that our method is robust to preprocessing noise, as we now note:

Furthermore, our method is fundamentally based on comparing collections of amino acid sequences via a distance rather than exact identity, which should make it more robust to sequencing error than methods based on exact matches of nucleotide sequence.

*The manuscript lacks comparison (at least a discussion thereof) of the described method with other methods for detecting repertoire difference (and even citation of them) beside Pogorelyy et al. PNAS 2018: for example, Dupic et al. PLOS Genetics 2021 (https://pubmed.ncbi.nlm.nih.gov/33395405/), Weber et al. bioRxiv 2022 (https://www.biorxiv.org/content/10.1101/2022.01.23.476436v1.full), Mayer-Blackwell elife 2021 (https://elifesciences.org/articles/68605), Slabodkin et al. Genome Research 2021 (https://genome.cshlp.org/content/early/2021/11/23/gr.275373.121.abstract), Alon Front Imm 2021 (https://www.ncbi.nlm.nih.gov/pmc/articles/PMC8047331/). Repertoire comparison is *the* main challenge of AIRR analysis. Please cite the literature appropriately.*

Thank you for the very comprehensive list of recent citations! We have updated the paper accordingly, including adding other related citations. All of these papers are related though different from the goals we have here.

The Slabodkin and Weber papers are based on various types of summary statistics (in the case of Slabodkin this summary statistic is the result of a model-fitting exercise).

The Alon and Dupic papers (along with the lovely Puelma Touzel paper which wasn't mention but now cited) all consider clones as individual "points" without a notion of similarity between them.

In constrast, the Mayer-Blackwell paper uses clustering based on sequence similarity to identify groups of

TCRs that may have similar binding properties rather than doing repertoire comparison as such (so in order to cite this paper we have brought in citations from other related work, all of which is a distinct line of work from what we are doing here).

The developed statistical test requires validation: the authors should show the p-value distribution in the case when the null hypothesis is true. And again, simulations would be nice as they would allow stress-testing the method.

From our perspective we are in an enviable and somewhat rare position for showing the effectiveness of our p-value analysis. Indeed, we wished to show that a simply computed null distribution using reshuffling is indicative of the result of running an independent experiment. What is remarkable about the data set analyzed in this paper is that we actually have replicate experiments (reflecting the true null distribution) with which we can perform a comparison. These data have all the characteristics of a true data set, including all the quirks that real data has. This makes our p-value comparison much stronger than one based on simulation.

We have modified wording in the following two sentences to make this more clear:

This data set has 23 sampled biological replicates for each cell type, which allows us to understand the true biological variability of observing a given TCR in a sample.

and then, later:

If the statistical characteristics of these true replicate loneliness distributions approximately match the statistical characteristics of the "randomization" loneliness distributions, this gives us confidence in our testing procedure and significance estimates as they perform on real data.

"We wished to develop a procedure that performs comparisons between two empirical repertoires in a fast, interpretable, and precise manner" -> where is the manuscript do you explain "interpretability"? The plots in the results section are overall very hard to understand (as is the text, please streamline), so it is not clear to us how this method can be practically used for repertoire comparison. We might also have a different definition of "comparison". It seems to us that your method is to point enriched clones versus a baseline? One can call this comparison, but one could have also been more direct as for example in this paper's title https://elifesciences.org/articles/68605.

There are two points here: first, interpretability, and second, comparison.

Regarding interpretability, the optimal transport method delivers a number that quantifies difference between repertoires, but more importantly provides a mapping between collections of TCRs that provides TCR-level differences between repertoires. This method is based on a biologically-motivated distance, TCRdist. Furthermore, our method returns sets of motifs that are characteristic of a given cluster of TCRs in comparison to others,

with motif visualizations that are output from the software. Putting these points together, we feel that this method qualifies our method as being "interpretable".

Regarding comparison, the method is fundamentally a means of doing detailed repertoire comparison: one puts two repertoires in and gets comparative information as described above. The reviewer is correct that in our application we have focused on finding enriched clones versus a baseline.

"While their predictions do not constitute the ground truth of actual responsive TCR clones to the YFV vaccination, they can still serve as a useful performance benchmark" -> why is another method a useful performance benchmark? And if the method by Pogorelyy is so useful as a benchmark, in what why is your method novel (or can bring about new biological insight which was not possible with Pogorelyy's method)?

First it may be worth clarifying that there are two excellent papers with Pogorelyy as first author from 2018. The paper described in our text here we might call the "YFV paper" as it was a specific analysis for understanding YFV-responsive clones. Because the statistical analysis for this paper was developed specifically for this data set, and requires longitudinal data, it seems fair to use the results of this analysis as our best chance of finding YFV-responsive clones.

The second Pogorelyy 2018 paper developed the ALICE method, which compares a single repertoire to a baseline generative model to identify collections of responsive clones. This paper used the YFV paper results as a benchmark for comparison, as we do here.

We have already described differences between our method and these other methods. Specifically, in comparison to the YFV paper our method does not require multiple longitudinal samples. In comparison to the ALICE method our method does not require a baseline generative model. In comparison to both methods, our method can be applied to compare two arbitrary repertoires.

Finally, in our revision we describe below a new simulation-based benchmark for which a ground truth can be confidently assigned.

Minor issues

The equations are denoted the same as references. Using "Eq." prefix for all equation references will make the text easier to read.

Equation references use round brackets while references use square brackets, but the difference may have been obscured by the hyperlink boxes inserted into the PDF.

Nevertheless, we have made the modification suggested by the reviewer.

Why is step size for clustering set to 5 by default?

This value was chosen somewhat arbitrarily based on the typical magnitude of TCRdist distances: a single non-conservative mismatch in a CDR1 or CDR2 loop increases TCRdist by 4 units, while a non-conservative mismatch in the CDR3 increases the TCRdist by 12 units. So total distances are often in the 10s - 100s, and a step size of 5 is not too big but also not too small.

The "Soldiers on a battlefield" example needs to be formalized a bit further: it is not entirely clear from the description whether we consider all soldiers different or not. More generally, do we really need a war analogy in scientific papers on TCRs?

We have clarified this analogy with:

In this analogy, each unit of probabilistic "mass" corresponds to a single distinct soldier, and higher probabilistic mass at a given location corresponds to a larger number of soldiers at that location.

Regarding the war analogy, we are entirely sympathetic, but the original motivating example was from the military as we now indicate with a citation to:

Vershik, A. M. (2013). Long History of the Monge-Kantorovich Transportation Problem. The Mathematical Intelligencer, 35(4), 1–9. https://doi.org/10.1007/s00283-013-9380-x

Code availability: it is said that all the code is available on Github but there is no link in the manuscript

We are truly sorry for this slip-up, which is ironic given the efforts we made towards documenting the work on the repository and making the analysis reproducible.

We have added an Implementation section to the manuscript, which points the reader to `https://github.com/matsengrp/transport`.

The more focused problem of inferring specificity from TCR sequences has been approached by tracking individual clones, comparing to a probabilistic model, or using epitope-specific machine-learning models. -> please provide citations for each claim.

We have added "as follows" to this sentence to clarify that it's an introductory topic sentence. More details, and citations, follow in the body of the paragraph.

Other machine learning techniques build predictive models using labeled training data [16–18], although these techniques often require a specified antigen epitope, can be limited by the amount of publicly-available data,

and rely on models that can be difficult to interpret. -> this sentence sounds like your approach will also be able to compare epitope-specific repertoires. Please rephrase.

Thank you for pointing out the need to clarify here: We have split this problematic section out to its own paragraph, as follows:

Another line of work uses experimentally-inferred antigen-associated TCRs as labeled data; this is a different goal than the one approached in this paper. For example, machine learning techniques can be used to build predictive models using these labeled training data [22-24]. These can be limited by the amount of publicly-available data, and rely on models that can be difficult to interpret. Another approach is to cluster sequences based on similarity [25-28].

For the datasets used, please mention how preprocessing was performed.

We have added the following statement concerning the IEL dataset:

The sequence data preprocessing for this study was performed using MIGEC [41]; more details can be found in [40].

The processed yellow fever data were downloaded from the web repository associated with the original study (`https://github.com/mptouzel/pogorelyy_et_al_2018`). We have added a statement to this effect in the manuscript.

Please define biological replicate.

We have clarified the meaning of this phrase in the data description:

The majority of our analyses involve TCRβ repertoires collected from 23 C57BL/6 mice [40], which form biological replicates.

Reviewer 2

Adaptive immune recognition relies on an incredibly diverse set of transmembrane receptors diversified through genetic recombination. The ability to read out this diversity through sequencing allows measurement of this diversity at unprecedented scale. To make good on the promise of repertoire sequencing to provide new insights into adaptive immunity, there is an important need for better statistical and computational analysis techniques for this complex data. In the paper 'Comparing T cell receptor repertoires using optimal transport', the authors propose using the mathematical framework of optimal transport as an elegant way of comparing similarity between T cell receptor repertoires. By building on recent advances in the field of optimal transport, namely the Sinkhorn distance formalism, the authors demonstrate computational tractability of the approach. Importantly,

the paper also provides some evidence that their approach can detect biologically meaningful differences between samples in case studies.

The proposal is conceptually innovative and the paper is well-written overall. However, as currently presented there are a number of concerns regarding the statistical foundations of the method and its benchmarking that should be suitably addressed.

Major concerns/comments/question:

Can the definition of the relative loneliness measure be given a less heuristic motivation? Or alternatively, can the consequences of this definition be better explored on a toy model? Are there any insights from theory into how to choose the neighborhood size delta?

Thank you for this suggestion. These are, indeed, somewhat challenging concepts to think about and explain. First, we have modified our terminology to enhance (we hope) clarity: "total loneliness" (Eq. 9) and "relative loneliness" (Eq. 10) have been renamed "individual loneliness" and "neighborhood loneliness" to clarify their differences. Also, our "spike-in" simulation study mentioned above nicely demonstrates that the neighborhood measure (Eq. 10) works much better than the per-TCR measure (Eq. 9) in highlighting the spiked-in TCRs that distinguish one repertoire from the other (Fig. S1).

The fit in Fig.6 has a slope significantly below one, which implies that the randomization z-scores consistently overestimate significance. This deserves explanation. Note that in applications where biological replicates are not available this severely limits the practical utility of the method.

The reviewer is correct that we should clearly state this, as we have now done:

We emphasize that the slope of this line is not one, and so the randomization p-value can not be interpreted directly as a significance test where the null is a biological replicate in a different individual organism. This is not surprising, since there is genuine biological variation between the various mice. If one desired to approximate a between-organism p-value, one could use the values of the linear regression to map the randomization Z-score to a replicate Z-score and calculate a p-value accordingly.

In the last results section, a more fair comparison would be with 1 CDR3aa mismatch as the number of true positives using this threshold is closer between both methods. The true positive to false positive ratio for the benchmark method is then 81/3 = 27. This implies a very different conclusion regarding the relative performance of the methods.

This is correct — thank you — and we have added the 1 CDR3aa mismatch analysis alongside the 2 CDR3aa mismatch one.

An important comparison to the ALICE method is missing from the current manuscript. Such a benchmarking is important as it is more direct than with the longitudinally identified sequences, as discussed by the authors. How well does the non-parametric method perform relative to ALICE, which uses additional information from its learned parametric model of recombination? An inferior performance might still make the simpler method presented here useful, but it is important to have an idea of how much statistical power is lost.

We have updated the manuscript with a simulation-based comparison with ALICE using a spike-in sample (see section "Simulation-based benchmarking").

Minor concerns/comments/questions:

The results only apply the loneliness measure to data, but in certain contexts the overall optimal transport distance might be interesting in its own right and could be illustrated in an application. A comparison and/or discussion of the optimal transport distance with distance measures that might be constructed from sequence-similarity weighted repertoire diversity measures might also improve the manuscript.

Thank you for the suggestion. We have added a tree built from the optimal transport score as Figure 6, illustrating the utility of this overall distance.

What is the biological motivation for analyzing outliers in DN with respect to CD4 in the first results section? Intuitively, the reverse comparison seems more biologically meaningful given the developmental lineage of T cells.

We suspect that there's a bit of confusion here, probably due to the "DN" terminology.

Quoting from the Schattgen et al paper from which these data are derived: "DN-IELs arise through a unique pathway of agonistic positive selection on transporter associated with antigen processing (TAP) and beta-2-microglobulin (β2m)-dependent self-antigens presented by MHC-Ia and other unidentified MHC-Ib molecules." These DN cells are distinct from the preselection "DN" cells in thymic development, being resident T cell subsets from the gut mucosa.

Thus, DN cells are unusual cells with properties we would like to better understand, motivating our comparison of DNs to more typical CD4 cells.

We have attempted to clarify this difference by adding to the sentence

In terms of receptors, the group of cells we call DN are CD4$^-$ CD8$\alpha\beta^-$ CD8$\alpha\alpha^+$, which are distinct from the class of preselection T cells which are sometimes also called "DN".

On line 125, it might help readability to define D in terms of x_i, y_j, as $D_{ij} = d(x_i, y_j)$.

Done, thanks!

The definition of the candidate set of TCRs and all sequences in any of the top10 clusters on lines 578-583 should be clarified. It remains unclear to me what precisely is meant by each.

Yes, thanks for the suggestion. We have broken this out to two sentences, like so:

We will say that a TCR for an individual is candidate-responsive if it is in a top-10 cluster (i.e. one of the top 10 highest ranked clusters by loneliness) for the individual's $+15$ day comparison. Define the candidate set *of responsive TCRs to be the union of candidate-responsive TCRs across the six individuals.*

The value of delta used in the results section should be indicated in the legend/text.

Thank you for pointing out this oversight! We have added the delta value along with a brief justification to the "Implementation" subsection.

A link to the github repository mentioned in the data/code availability statement should be added.

You are absolutely right, and sorry for the omission. We have included it.

Typos:

line 159: $\mathbf{ab}^T$ -> $\mathbf{rc}^T$

Whoops, thanks!

*line 274: to *be* estimated*

Fixed, thanks!

Reviewer 3

This study introduces a clever strategy to compare and analyze TCR repertoire distributions using the optimal transport method. The advantage that this strategy offers over the probability generation models that identify unique clusters in a given repertoire is that it uses a combination of probability mass distribution and the similarity-matched distance metric to identify uniquely enriched TCR sequences in a repertoire under consideration as compared to a reference repertoire. The study provides sufficient explanation of the working principle of the method and the evidence of its applicability to publicly available datasets. The independence from modelling assumptions and parametric approximations can be viewed as the strength, however the success of this approach heavily relies on the quality of the data and availability of a compatible reference repertoire. It may be helpful if authors highlight the unique insights that may emerge from using this method to compare TCR distributions (e.g. see major point 2 below), in addition to focussing on benchmarking its performance in comparison to other studies.

Major points:

The major concern using a reference repertoire to gain insights about a test repertoire is context dependency. For example, it is possible that a clone responding to a vaccination strategy is also enriched in the pre-vaccination reference repertoire due to either high probability of generation (and peripheral selection) or due to prior immunization experience. Such clone would not be picked up as "lonely" using this approach. Conversely, a relatively weakly responding clone may have a very low abundance in the reference repertoire and thus would rank high on the lonely scale. How does this approach handles such clonal abundance disparity?

Thank you for the opportunity to clarify this point. We have added:

By default, each independent input TCR sequence is given equal probability mass in the transport analysis. For input files that have been reduced to unique nucleotide-level clonotypes, as in the present manuscript, this has the effect of ignoring clonal abundance (except when subsetting to the top expanded clonotypes, as in the YFV analysis). The extent of clonal abundance (i.e., the numerical sizes of expanded clonotypes) can be noisy and sequencing-method dependent, which makes this a more conservative approach: significant differences in TCR landscape density are driven by accumulation of independent rearrangement/selection events rather than individual clonal expansions. For situations in which one wants to use clonal abundance, it is straightforward to re-duplicate clonotypes prior to input to the pipeline, which will assign additional mass proportional to the number of copies of each clonotype.

This approach may also allow for the analysis and quantification of inter-individual variability in the immune

responses. For example, the authors could compare post vaccination d15 TCR repertoires of individuals immunized with yellow fever vaccine, which may reveal valuable insights about the differences in clonal distributions and how they affect the dynamics of response to the vaccine. Especially, in individuals with high and low hit rates between 0d and 15d comparisons (P1 and Q1, for example).

This is an excellent suggestion for future work. We agree that the optimal transport framework may allow for deeper analysis of inter-individual variability, however we believe that this analysis would be more appropriate for a follow-up study.

The definitions of $w(c)$ *and AAdist need justification (Lines 183 and 184).*

These are part of the TCRdist algorithm, which was published in 2017 and has been cited almost 500 times.

We now note that these formulas are justified in the methods section of the paper defining TCRdist, in the section "TCR distance measure."

Why do authors say that predictions from Pogorelyy et al. do not constitute the ground truth of actual responsive TCR clones to YFV vaccination. Please justify respectfully. (Line 545).

The Pogorelyy method uses a statistical model to find responsive clones based on sampled longitudinal frequency. Although this is an excellent method, any such method is subject to false positives and negatives due to sampling stochasticity. Furthermore, a clone may be responsive but at a low enough level so as not to be detectable above noise. Indeed, Pogorelyy et al themselves feel the need to benchmark their inferential method by comparing it to the VDJdb database, which is a curated database of TCRs with experimentally-characterized specificities.

During clustering, does the Algorithm 1 reach the breakpoint because no more sequences are found in increasing radii or because too many sequences are added to the cluster such that the effective decrease in the mean loneliness is substantially small?

It may be helpful to refer to Fig. S1, which shows the scatterplots that are used to find the breakpoints. As the clustering radius increases, the mean loneliness goes down because we are adding additional sequences that are less lonely than the original most-lonely sequence. At some point this decrease levels off and we reach the breakpoint.

So to specifically answer the question, no, we aren't running out of sequences, but rather we are progressively leaving our cluster of lonely sequences.

If the reviewer missed Fig. S1, perhaps that's not surprising because we forgot to reference the figure in the manuscript. We now refer to them directly by saying:

To gain intuition about how the algorithm works, and as a visual confirmation of the assumptions behind the algorithm, we refer the reader to Fig. S1.

Minor points:

Discussion of the productivity-based filters that limit the diversity of circulating pool of TCR sequences is needed. (Line 9-10).

We have expanded to

Even after a series of productivity-based filters ensuring functionality and limiting self-reactivity

and added a citation. We feel that doing more would distract us from the purpose of this introduction.

Equation numbering needs to be careful and consistent. Some equations are referred in the text as Eq. XX while some are just referred (XX). Also line 452 should be Eq. 18.

We have added the "Eq" prefix everywhere as requested by Reviewer 1.

Thanks for the fix on line 452!

Define "hit rate" properly (line 549).

We have expanded this as follows:

In particular, we define the "hit rate" as the empirical probability that a clone our procedure detects as responsive was also detected by the original authors as responsive, i.e. the number of sequences we detected as responsive that were in the original responsive set, divided by the number of clones our procedure detects as responsive. We can explore how this "hit rate" varies by timepoint, cluster rank, and donor.
